# Twinning-Induced Abnormal Strain Rate Sensitivity and Indentation Creep Behavior in Nanocrystalline Mg Alloy

**DOI:** 10.3390/ma14227104

**Published:** 2021-11-22

**Authors:** Shilun Yu, Yingchun Wan, Chuming Liu, Zhiyong Chen, Xiangyang Zhou

**Affiliations:** 1 School of Metallurgy and Environment, Central South University, Changsha 410083, China; yushilun@csu.edu.cn; 2 Light Alloy Research Institute, Central South University, Changsha 410083, China; 3 School of Materials Science and Engineering, Central South University, Changsha 410083, China; cmliu803@sina.com (C.L.); czysh@netease.com (Z.C.)

**Keywords:** Mg alloy, nanocrystalline, nanoindentation, strain rate sensitivity, creep

## Abstract

Nanocrystalline materials exhibit many unique physical and chemical properties with respect to their coarse-grained counterparts due to the high volume fraction of grain boundaries. Research interests on nanocrystalline materials around the world have been lasting over the past decades. In this study, we explored the room temperature strain rate sensitivity and creep behavior of the nanocrystalline Mg–Gd–Y–Zr alloy by using a nanoindentation technique. Results showed that the hardness and creep displacements of the nanocrystalline Mg–Gd–Y–Zr alloy decreased with increasing loading strain rate. That is, the nanocrystalline Mg–Gd–Y–Zr alloy showed negative strain rate sensitivity and its creep behavior also exhibited negative rate dependence. It was revealed that the enhanced twinning activities at higher loading strain rates resulted in reduced hardness and creep displacements. The dominant creep mechanism of the nanocrystalline Mg–Gd–Y–Zr alloy is discussed based on a work-of-indentation theory in this paper.

## 1. Introduction

Mg alloys are promising metallic structural materials and regarded as ideal candidates for lightweight applications in automotive and aerospace industries owing to their low density, high specific strength and rich mineral resources on earth [1,2]. However, the low absolute strength compared with Al alloys limits the wide application of Mg alloys. Alloying with rare earth (RE) elements is proven effective in fabricating high-performance Mg alloys. Among various Mg–RE alloys, the Mg–Gd–Y series alloys are attracting increasing attention due to their excellent combination of tensile strength, ductility, and creep resistance [3,4,5,6,7,8]. Homma et al. [9] reported an extraordinary high-strength Mg–Gd–Y–Zr alloy with an ultimate tensile strength of 542 MPa, yield strength of 473 MPa, and elongation of 8%. Grain refinement via severe plastic deformation is an effective way to strengthen metallic materials [10,11], which has been successfully applied to many face-centered cubic (fcc) [12,13], body-centered cubic (bcc) [14,15], and hexagonal close-packed (hcp) metals and alloys [3,6,16,17]. Using room temperature rotary swaging, Wan et al. [8] successfully fabricated a bulk nanocrystalline (NC) Mg–Gd–Y–Zr alloy which possesses an average grain size of 80 nm and exhibits an ultrahigh ultimate tensile strength and yield strength of 710 MPa and 650 MPa, respectively. This work sheds light on the potential in strengthening Mg alloys via grain refinement. Sun et al. [6] achieved a hardness as high as 145 HV in a nanostructured Mg-8.2Gd-3.8Y-1.0Zn-0.4Zr alloy that was prepared using high-pressure torsion (HPT).

Previous studies have demonstrated that when grain sizes are refined to nanoscale, most metals and alloys can obtain double or even much higher strength with respect to their coarse-grained (CG) counterparts [18,19,20]. With grain sizes entering nanoscale, the volume faction of grain boundaries (GBs) increases dramatically. Besides the unprecedented high strength of NC metals and alloys, there are many other GB-related phenomena that are enhanced in NC materials, such as GB sliding, grain rotation, and Coble creep [20,21,22]. Owing to these special characteristics, NC materials usually show many different ambient or elevated temperature deformation mechanisms as compared with their CG counterparts.

The strain rate sensitivity (SRS) index, *m*, is a crucial parameter which reflects the underlying deformation mechanisms of metallic materials during plastic deformation. Extensive investigations have been conducted on the SRS of NC materials and the relationship between *m* and grain sizes of crystalline materials [23,24,25,26]. Generally, plastic deformation of conventional CG materials proceeds via dislocation slip and interaction, which correspond to a relatively small *m* value. As grain sizes decrease, the volume fraction of GBs increases and GB-mediated mechanisms are enhanced, leading to a larger *m*. For example, when superplastic deformation (GB sliding) occurs, *m* is close to 0.5 [27]; when Coble creep (GB diffusion) dominates the plastic deformation, *m* theoretically equals 1 [28]. This variation tendency applies well to fcc metals, such as Al [29], Cu [25,26], and Ni [24,30]. Ma [24] reported that the *m* value of NC Ni (*d* = 30 nm) is about four times that of CG Ni. However, studies by Wang et al. [31] and Wei et al. [25] suggested that the correlation between *m* and grain sizes depends on crystal structure. While *m* increases with decreasing grain sizes for fcc metals, it decreases with decreasing grain sizes for bcc metals. Taking Fe as an example, *m* decreases from ~0.054 for CG Fe [14] to ~0.009 for NC Fe [25]. Wei et al. [25] attributed the smaller *m* of NC bcc metals to the lower mobility of screw dislocations as grain sizes enter nanoscale. By far, most studies about the SRS of NC materials are focused on materials with cubic structures. Little information can be obtained about NC materials with hcp structure, such as Mg alloys. One purpose of the present work is to understand the deformation mechanism of the NC Mg alloy through examining its SRS at room temperature.

Creep is a crucial in-service deformation mode in engineering materials. Considering practical engineering applications, creep performance is an important factor that needs to be evaluated. Due to the enhancement of GB-related activities in NC materials, their creep behaviors may significantly differ from those in their CG counterparts. Studies on the creep behaviors of NC materials, such as Cu [32,33], Al [34,35], Ni [36,37], etc., indicate that the dominant creep mechanisms for most NC materials are GB-mediated processes. Moreover, due to the enhancement of GB-related activities, NC materials even creep at ambient temperatures at a relatively fast rate, which might accelerate material failure. Studies on many NC materials have demonstrated the existence of room temperature creep phenomena [38,39]. Therefore, although conventional CG Mg–RE series alloys are well developed creep-resistant materials [4,5,40], creep might occur at room temperature when their grains are refined into nanoscale. Taking the NC Mg–Gd–Y–Zr alloy as model material to explore its room temperature creep behavior is the second purpose of the present work.

A nanoindentation test is an effective and high-efficiency method to characterize the SRS and creep behaviors of NC materials [41,42]. Here, we explored the room temperature SRS and indentation creep behavior of a NC Mg–Gd–Y–Zr alloy by using a nanoindentation technique. Results indicated that both the hardness and creep displacement of the NC Mg alloy decrease with increasing loading strain rate. That is, the NC Mg alloy shows negative SRS and its creep behavior exhibits negative rate dependence.

## 2. Materials and Methods

### 2.1. Materials Preparation

Initial alloy ingot used in the present work was produced using semicontinuous casting, with a measured chemical composition of Mg-6.0Gd-3.5Y-0.5Zr (wt%). After solid solution treatment at 510 °C for 16 h, the billets with dimensions of Φ115 mm × 200 mm were extruded to Φ18.2 mm rods at 400 °C. Subsequently, the extruded rods were rotary swaged to Φ14.7 mm by four passes at room temperature, with a total area reduction of 34.8% [7,8].

### 2.2. Materials Characterization

Microstructural observations were performed on a FEI Helios NanoLab 600i dual beam scanning electron microscope (SEM) (Hillsboro, OR, USA) equipped with an Oxford electron backscatter diffraction (EBSD) system (Oxford, UK) and FEI Titan G^2^ 60-300 transmission electron microscope (TEM) (Hillsboro, OR, USA) operated at 300 kV. EBSD samples were prepared by electropolishing in ethanol solution containing 5 vol% perchloric acid at −40 ℃. EBSD data were analyzed using HKL Channel 5 software (Oxford, UK). TEM samples were mechanically ground to ~40 μm and then ion milled to perforation using a Gatan 691 precision ion polishing system (Pleasanton, CA, USA). X-ray diffraction (XRD) was conducted on a Bruker D8 Advance diffractometer (Karlsruhe, Germany) using CuKα radiation (λ = 0.154 nm) with a scanning step size of 0.02°, a counting time of 3 s, and a 2θ range of 10–80°.

**Figure 1:**
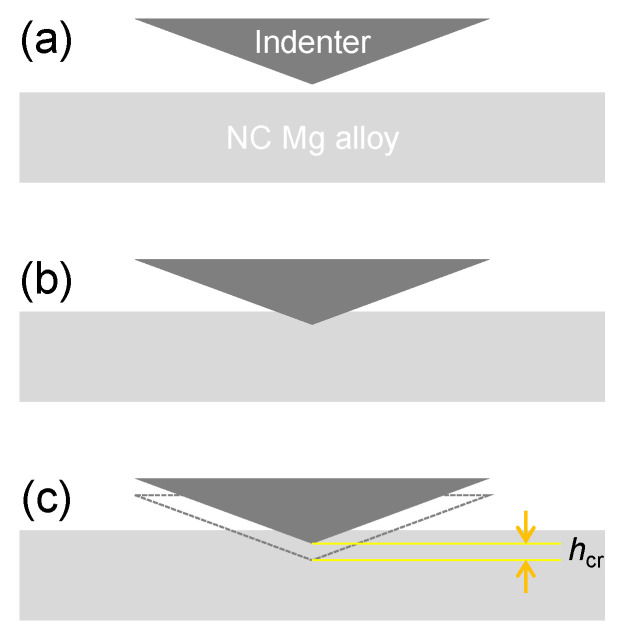
Diagram showing the interaction between indenter and material surface: (**a**) before loading, (**b**) during loading, and (**c**) holding stage.

Hardness and indentation creep behavior were characterized using an Anton Paar NHT 3 Nanoindenter (Graz, Austria) with a load and displacement resolution of 0.01 μN and 0.01 nm, respectively. Nanoindentation tests were conducted at loading strain rates (LSRs) ranging from 5 × 10^−3^ s^−1^ to 1 s^−1^, loaded to a predetermined depth of 2000 nm and held at corresponding maximum loads for 1500 s. Figure 1 shows the interaction between the indenter and the surface of the NC Mg alloy, in which *h*_cr_ represents the creep displacement during the holding stage (Figure 1c). For each set of experimental parameters, the tests were repeated five times to ensure data reliability. Samples for nanoindentation tests were mechanically ground and polished to mirror finish. Except for cases specified, all above-mentioned tests were performed on the cross sections of the rods.

### 2.3. Conventional Theory of Creep Stress Exponent Calculation

According to the classical power-law creep theory, creep stress exponent *n*, can be defined as [43]:

(1) $$n=\frac{\partial \log\dot{\varepsilon }}{\partial \log\sigma }$$

where $\dot{\varepsilon }$ is a steady state creep strain rate corresponding to stress *σ*. For indentation creep, the displacement time (*h*-*t*) curves during the holding stage can be fitted using an empirical equation [44]:

(2) $$h=h_{0}+a{\left(t-t_{0}\right)}^{b}+kt$$

where *h*_0_, *a*, *t*_0_, *b* and *k* are fitting parameters. Based on Equation (2), creep strain rate $\dot{\varepsilon }$ can be defined as [45]:

(3) $$\dot{\varepsilon }=\frac{1}{h}\frac{dh}{dt}$$

where *h* is an instantaneous indentation depth, and *t* is creep time. Creep stress *σ* during the holding stage can be obtained via Tabor relation, *σ = H*/3 [46], and the hardness *H* can be calculated by:

(4) $$H=\frac{F}{ch_{c}^{2}}$$

where *F* is real time load during the holding stage, *h*_c_ is contact depth, and *c* = 24.56 for the Berkovich indenter [45]. Combining Equations (1)–(4), creep stress exponent *n* can be obtained by plotting $\dot{\varepsilon }$ versus *H* in a double logarithmic coordinate system. The fitting slop of the linear segment of the log$\dot{\varepsilon }$-log*H* curve equals the creep stress exponent *n*.

## 3. Results

### 3.1. Microstructure

**Figure 2:**
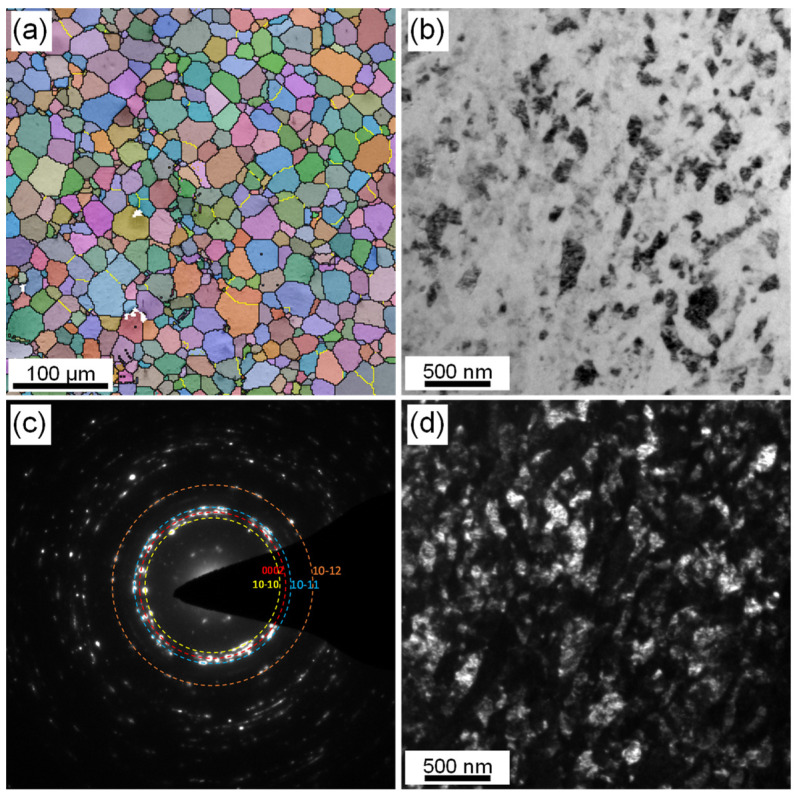
(**a**) EBSD Euler map of the coarse-grained Mg–Gd–Y–Zr alloy; (**b**–**d**) TEM bright field, SAED, and dark field images of the nanocrystalline Mg–Gd–Y–Zr alloy.

**Figure 3:**
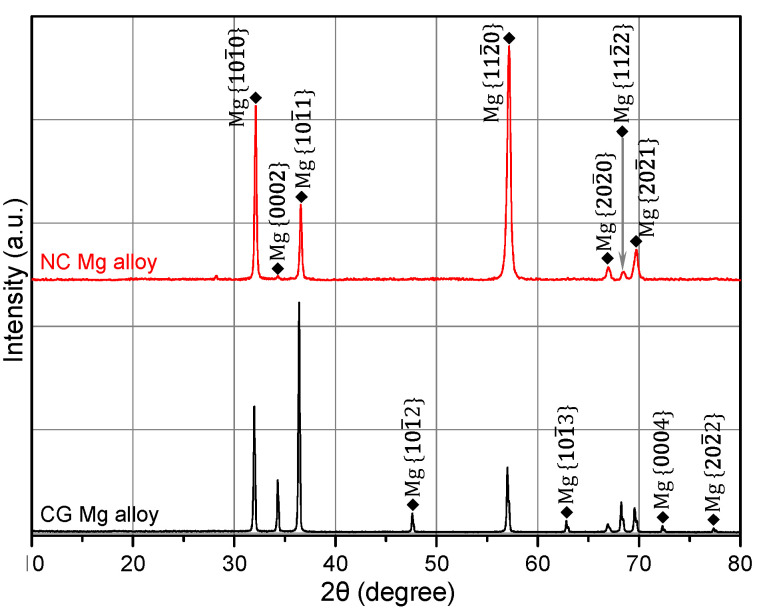
XRD patterns of the cross sections of nanocrystalline (NC) and coarse-grained (CG) Mg–Gd–Y–Zr alloys.

Figure 2 shows the microstructure in the central areas of extruded CG and swaged NC Mg–Gd–Y–Zr alloy rods. The initial extruded CG Mg alloy rod possessed a fully recrystallized equiaxed microstructure, with an average grain size of 17.1 μm (Figure 2a). After four passes of rotary swaging (RS), the microstructure in the central area of extruded CG alloy rod has been fully nanocrystallized, with its average grain size refined from 17.1 μm to 57.8 nm, as shown in Figure 2b–d. XRD patterns of the cross sections of CG and NC Mg alloy rods, as shown in Figure 3, suggest that the alloy is a single-phase solid solution before and after RS deformation. Moreover, the XRD profile of the alloy showed an obvious change after RS deformation. The diffraction peak of {0002} almost disappeared, accompanied by the enhancement of {10$\bar{1}$0} and {11$\bar{2}$0} diffraction. The sharp {10$\bar{1}$0}, {11$\bar{2}$0}, and extremely weak {0002} peaks indicated that the as-prepared NC Mg alloy possessed a strong basal fiber texture, with most of its grains oriented in such a way that their {0002} crystal planes are parallel to the axial direction of the swaged rod.

### 3.2. Strain Rate Sensitivity

**Figure 4:**
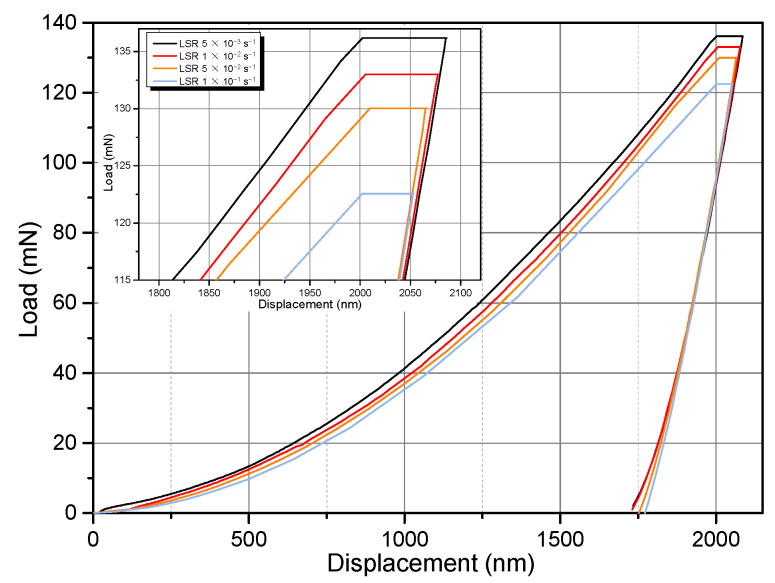
Load-displacement curves of the RS NC Mg–Gd–Y–Zr alloy under various loading strain rates.

**Figure 5:**
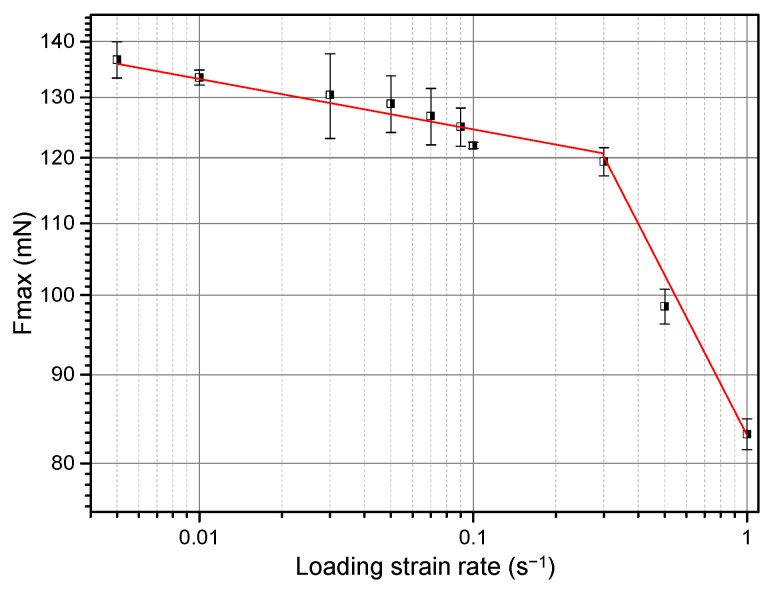
Relationship between maximum holding loads *F*_max_ and loading strain rates of the RS NC Mg–Gd–Y–Zr alloy.

**Figure 6:**
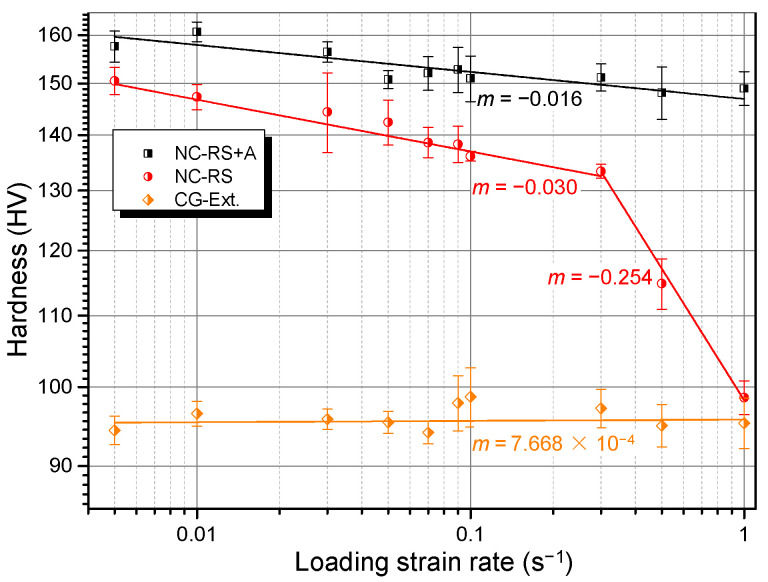
Room temperature strain rate sensitivity of NC and CG Mg–Gd–Y–Zr alloys.

Table 1 lists all the samples that were examined in this work. Figure 4 shows four exemplary nanoindentation load-displacement curves of the RS NC Mg alloy loaded perpendicular to the cross section. Unexpectedly, with increasing LSR, the maximum holding load, *F*_max_, decreased monotonously. That is, it exhibited negative strain rate dependence. Figure 5 summarizes the *F*_max_ corresponding to all the tested LSRs. *F*_max_ decreased from 136.7 mN at 5 × 10^−3^ s^−1^ to 83.2 mN at 1 s^−1^. Plotted in Figure 6 is the rate dependence of hardness of RS and aged (200 °C/18 h, designated as RS+A hereafter) NC Mg alloys in the double logarithmic coordinate system, in which the linear fitting slope is SRS index *m*. For comparison, SRS of the extruded CG alloy is also shown in Figure 6. While the CG Mg alloy exhibits positive but virtually negligible *m*, the NC Mg alloys show unexpected negative *m* values, which is different from those of most CG and NC materials [14,24,25,26,29,30,31]. A saltation at LSR = 3 × 10^−1^ s^−1^ in the SRS of NC–RS sample existed. Its *m* value decreased abruptly from −0.030 to −0.254. However, the saltation disappeared in NC–RS+A sample. Moreover, the *m* value increased to −0.016 after ageing treatment.

**Figure 7:**
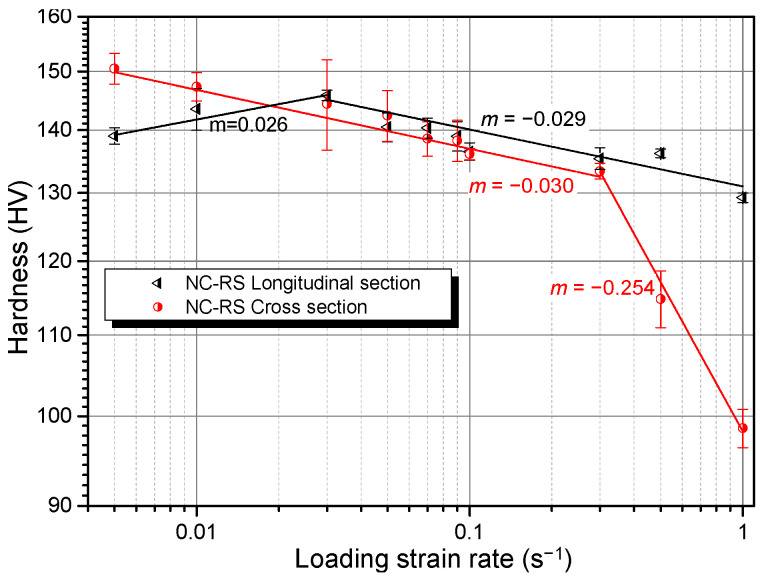
Room temperature strain rate sensitivity of cross and longitudinal sections of the NC Mg–Gd–Y–Zr alloy.

For materials with specific texture, the change of loading direction may influence their dominant deformation modes, which would therefore result in the variation of SRS. Figure 7 exhibits the SRS of the NC–RS sample loaded on different sections. As expected, the SRS of the longitudinal section showed different features with respect to the cross section. When the LSR < 3 × 10^−2^ s^−1^ the *m* value of the longitudinal section is positive and when 3 × 10^−2^ s^−1^ < LSR < 3 × 10^−1^ s^−1^, both sections possess similar *m* values; when LSR > 3 × 10^−1^ s^−1^, *m* of longitudinal section maintains −0.029, while that of cross section decreases suddenly to −0.254. Abnormal SRS undoubtedly stems from specific deformation mechanisms. The variation of *m* with LSR should be correlated to the transition of dominant deformation modes, which will be discussed in detail in Section 4.

### 3.3. Indentation Creep Behavior

#### 3.3.1. Rate Dependence of Creep Displacement

**Figure 8:**
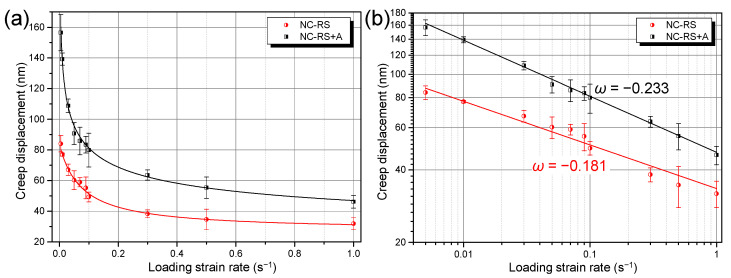
Strain rate dependence of room temperature indentation creep displacements of the NC Mg–Gd–Y–Zr alloys in (**a**) linear and (**b**) double logarithmic coordinate system.

Figure 4 implies that the displacements during holding stage, i.e., creep displacements, decrease gradually with increasing LSR. Figure 8a summarizes the creep displacements under ten LSRs. Attention should be paid to two characteristics. First, within the tested LSR range, creep displacements of the NC Mg alloy decreased monotonously with increasing LSR. Second, the ageing treatment surprisingly weakened the creep resistance of the alloy, as demonstrated by the larger creep displacements of the NC–RS+A sample. Referring to the definition of the SRS index *m*, here we define the strain rate dependence of indentation creep displacement as *ω* using Equation (5):

(5) $$\omega =\frac{\partial \log h_{\mathrm{cr}}}{\partial \log{\dot{\varepsilon }}_{L}}$$

where *h*_cr_ is the indentation creep displacement, and ${\dot{\varepsilon }}_{L}$ is the LSR. Figure 8b shows that the *ω* of both RS and the RS+A NC alloys are negative. That is, not only the hardness, but the creep displacements of the NC Mg alloys exhibit negative strain rate dependence as well.

**Figure 9:**
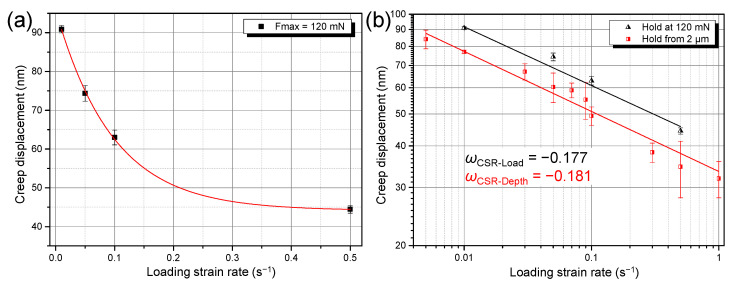
Strain rate dependence of room temperature indentation creep displacements of the RS NC Mg–Gd–Y–Zr alloys at fixed *F*_max_ in (**a**) linear and (**b**) double logarithmic coordinate system.

The aforementioned results were obtained based on the mode that the indenter was loaded to a fixed depth, 2000 nm, at a constant strain rate followed by holding at the corresponding maximum load (designated as CSR-depth mode hereafter). Under such a loading mode, the holding load, *F*_max_, decreases with increasing LSR in the present work, as shown in Figure 5. It is therefore reasonable to question whether the reduced creep displacements at higher LSRs are caused by reduced holding loads. To answer this, we designed a set of control experiments. The indenter was loaded to a fixed load, 120 mN, at a constant strain rate, followed by holding at 120 mN for the same duration as in the CSR-depth mode (designated as CSR-load mode hereafter). Figure 9 gives the results of the CSR-load experiments. It is apparent that the creep displacements still decreased with increasing LSR, which follows the same variation tendency as in the CSR-depth mode (see Figure 9b). This result rules out the possibility that the lower holding loads at higher LSRs lead to lower creep displacements. Instead, it verifies the fact that the negative strain rate dependence of indentation creep displacements is an intrinsic property of the NC Mg–Gd–Y–Zr alloy in the present work.

#### 3.3.2. Creep Mechanism

**Figure 10:**
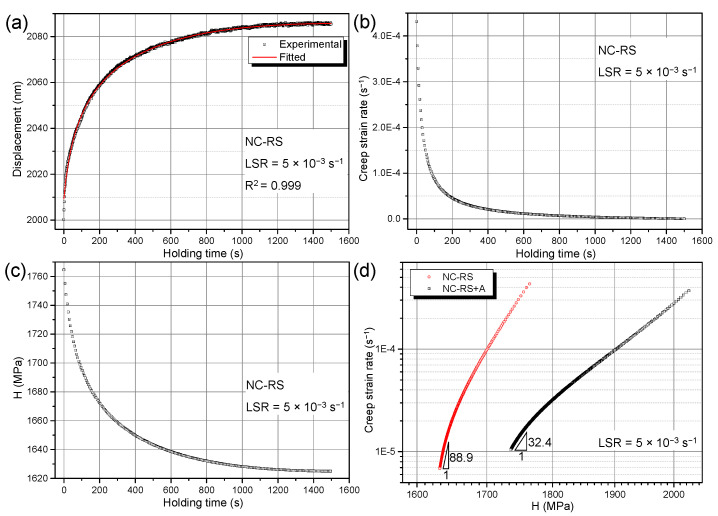
General procedures for calculating creep stress exponent *n* via nanoindentation tests: (**a**) *h*-*t* curve; (**b**) $\dot{\varepsilon }$-*t* curve; (**c**) *H*-*t* curve; (**d**) log$\dot{\varepsilon }$-log*H* curves.

Creep stress exponent *n* is an important parameter in describing the creep process, and its value reflects the creep mechanisms of materials. Generally, *n* = 1 corresponds to diffusion creep, *n* = 2 corresponds to GB sliding, and *n* = 3–7 corresponds to dislocation creep [43]. Figure 10 elaborates the detailed calculation process of *n* based on conventional theory. Taking LSR = 5 × 10^−3^ s^−1^ as an example, fitting using Equation (2) gives an excellent agreement with the experimental result, as shown in Figure 10a. Based on Equation (3), the $\dot{\varepsilon }$-*t* curve corresponding to Figure 10a can be obtained, as shown in Figure 10b. As holding time exceeded ~1000 s, $\dot{\varepsilon }$ decreased very slowly, indicating the arrival of a steady state creep. Based on Equation (4), one can obtain the *H*-*t* curve corresponding to Figure 10a, as shown in Figure 10c. Combining Equations (1)–(4), the creep stress exponent *n* can be obtained by plotting versus *H* in a double logarithmic coordinate system. Figure 10d shows the log$\dot{\varepsilon }$-log*H* curves for RS and the RS+A NC Mg alloys, in which the *n* values of RS and the RS+A NC Mg alloys are 88.9 and 32.4, respectively. Similar to the results of most indentation creep studies on metals and alloys, the *n* values obtained via conventional data processing procedures were considerably larger than the range of 1–7 corresponding to the classical power-law creep theory. It is impossible to accurately analyze creep mechanisms based on such abnormally large *n* values.

To overcome this difficulty, a redefined creep strain rate and indentation creep hardness were put forward based on the concept of work of indentation [47,48,49]. According to the work-of-indentation theory by Stilwell and Tabor [50], hardness obtained via nanoindentation tests can be defined as:

(6) $$H_{\mathrm{WI}}=\frac{W_{p}}{V_{p}}$$

where *H*_WI_ is hardness based on work-of-indentation theory, *W*_p_ is plastic work conducted by the indenter, and *V*_p_ is plastically deformed volume. Referring to the definition of *H*_WI_, indentation creep hardness, *H*_cr_, can be defined as:

(7) $$H_{\mathrm{cr}}=\frac{W_{\mathrm{cr}}}{∆V_{\mathrm{cr}}}$$

where *W*_cr_ is the plastic work conducted by the indenter during the creep stage, and $∆V_{\mathrm{cr}}$ is the variation of the plastically deformed volume during creep stage. *W*_cr_ and $∆V_{\mathrm{cr}}$ can be calculated using Equations (8) and (9):

(8) $$W_{\mathrm{cr}}=F_{m}\left(h_{m}-h_{0}\right)$$

(9) $$∆V_{\mathrm{cr}}=\frac{c}{3}(h_{m}^{3}\_h_{0}^{3})$$

where *F*_m_ is holding load, *h*_0_ and *h*_m_ are displacements at the start and end of holding stage, respectively, and *c* = 24.56 for the Berkovich indenter [45]. Since creep-induced $∆V_{\mathrm{cr}}$ is very small with respect to *V*_p_, it is reasonable to treat *V*_p_ as constant during the holding stage. Therefore, the creep strain rate based on work-of-indentation theory, can be formulated as:

(10) $${\dot{\varepsilon }}_{\mathrm{cr}}=\frac{∆V_{\mathrm{cr}}}{V_{p}}\frac{1}{∆t}$$

where Δ*t* is the holding duration. According to the hemisphere hypothesis, the plastically deformed volume *V*_p_ can be obtained as follows [51]:

(11) $$V_{p}=\frac{1}{2}\cdot \frac{4}{3}\pi r^{3}$$

where the radius of hemispherical plastically deformed volume *r* can be expressed as [49]:

(12) $$r={\left(\frac{3F_{m}}{2\pi \sigma_{s}}\right)}^{1/2}$$

where *σ*_s_ is yield strength and can be obtained via Tabor relation, *σ*_s_ = *H*_p_/3. Based on the work-of-indentation theory, Tuck et al. [48] related *H*_p_ to holding load *F*_m_ and plastic work *W*_p_ using Equation (13):

(13) $$H_{p}=\frac{\kappa F_{m}^{3}}{9W_{p}^{2}}$$

where κ is an indenter shape-dependent constant. For the Berkovich indenter, κ = 0.0408 [48]. Referring to the definition of *n* in Equation (1), the creep stress exponent based on the work-of-indentation theory *n*_cr_ can be expressed as:

(14) $$n_{\mathrm{cr}}=\frac{\partial \log{\dot{\varepsilon }}_{\mathrm{cr}}}{\partial \log H_{\mathrm{cr}}}$$

**Figure 11:**
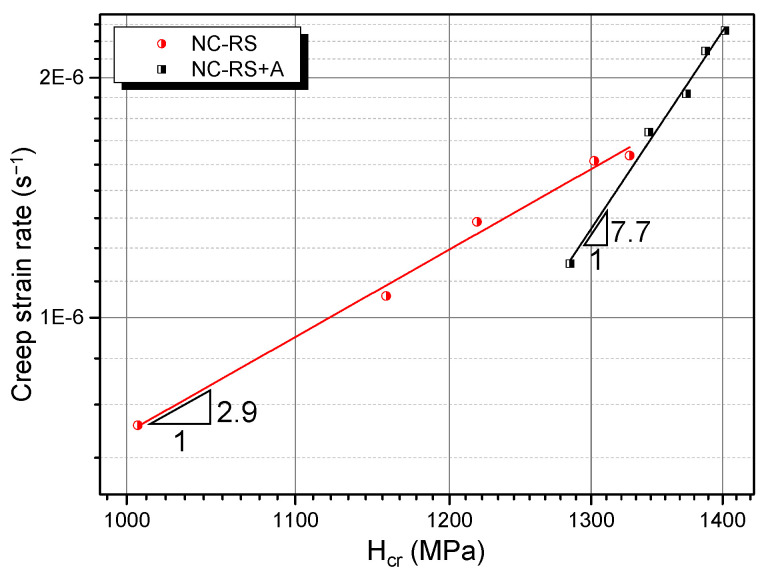
Calculation of the creep stress exponent based on the work-of-indentation theory.

Combining Equations (7)–(13), *H*_cr_ and ${\dot{\varepsilon }}_{\mathrm{cr}}$ corresponding to each LSR can be obtained. According to Equation (14), one can deduce *n*_cr_ by plotting ${\dot{\varepsilon }}_{\mathrm{cr}}$ versus *H*_cr_ obtained above in a double logarithmic coordinate system, as shown in Figure 11. Creep stress exponents of the NC Mg alloys obtained via different data processing approaches are summarized in Table 2. It is clear that the results based on the work-of-indentation theory are much smaller and agree with the classical power-law creep. Values of *n*_cr_ suggest that room temperature indentation creep mechanisms of RS and RS+A NC Mg alloys are a dislocation creep. For the former, owing to the existence of supersaturated solute atoms inside grains, the dislocation glide is dragged by the solute atmosphere, resulting in a dislocation viscous glide creep mechanism (*n*_cr_ = 2.9) [43]. For the latter, the supersaturated solid solution decomposed during the ageing treatment, forming solute clusters or grain boundary solute segregations [7], which reduced the solute concentration in the *α*-Mg matrix and therefore weakened the solute drag effect. Hence, the dislocation climb becomes the rate-controlling step during the room temperature creep of RS+A NC Mg alloy (*n*_cr_ = 7.7) [43].

## 4. Discussion

### 4.1. Negative Strain Rate Sensitivity

Generally, metals and alloys become harder at higher strain rates due to enhanced dislocation accumulations, known as usual positive SRS (PSRS). However, under some specific circumstances, materials can soften at higher strain rates, showing a phenomenon of negative SRS (NSRS). By far, there are four mechanisms that can explain the NSRS phenomenon: dynamic strain ageing (DSA) [52,53,54,55], stress-induced phase transformation (SIPT) [56,57], formation of microcracks [54,58], and enhanced twinning activities at higher strain rates [59,60,61].

DSA works via solute-dislocation interactions [62]. At relatively lower strain rates, solute atoms can segregate onto mobile dislocations, increasing their motion resistance. With increasing strain rate, the amount of solute atoms that can follow mobile dislocations decreases, reducing motion resistance and leading to NSRS. Thus, at a fixed temperature, DSA can only work below a critical strain rate, beyond which solute atoms can no longer follow dislocations and dislocation-accumulation-induced PSRS takes over [54,55]. It is therefore appropriate to claim that the NSRS of the NC Mg alloy in the present work is not caused by the DSA effect for the following reasons. First, the *m* value for the cross section remains negative under all tested strain rates, and not only does it not increase gradually with increasing strain rate, but it decreases abruptly when LSR > 3 × 10^−1^ s^−1^ (see Figure 7). Meanwhile, the result on the longitudinal section indicates that it shows NSRS at higher strain rates, but exhibits PSRS at lower strain rates (see Figure 7), which is opposite of the features of DSA. Second, the stress-strain curves of the Mg–Gd–Y–Zr alloy used in the present work (not shown here) do not show serrated flow behavior, a typical characteristic induced by the DSA effect [52,53,63]. This is owing to the fact that Gd and/or Y atoms diffuse slowly in the Mg matrix and cannot follow mobile dislocations [64], while the ability to follow dislocations is a prerequisite for the occurrence of DSA.

SIPT commonly lead to NSRS in materials that may experience phase transformation under applied stress, such as *β*-Ta [56], 304 austenitic steel [65], and Ti–10V–2Fe–3Al alloy [57]. Obviously, the prerequisite for the occurrence of SIPT-induced NSRS is the polymorphism of the investigated materials. However, except for the Mg–Li alloy [66], there is no report claiming the discovery of a new crystal structure in Mg alloys in addition to hcp structure by far. Thus, the possibility of SIPT is also ruled out.

**Figure 12:**
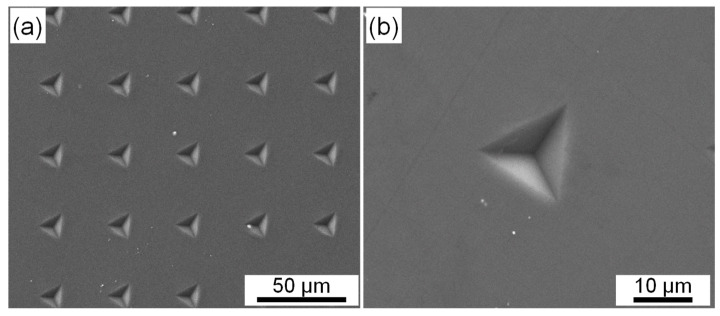
SEM images of indentation morphology showing no microcrack: (**a**) low and (**b**) high magnification.

Recently, studies on metallic multilayers suggests that the propensity of crack formation increases at higher strain rates, and this can result in NSRS [58]. This such mechanism does not apply to the present situation considering the nature of bulk single-phase material other than multilayers. Moreover, SEM inspection of residual indentations verifies that all the indentations are intact, with no microcrack (see Figure 12).

It is well-known that twinning propensity is enhanced at higher strain rates [67]. Therefore, under circumstances where the dislocation slip is suppressed or saturated, enhanced twinning activities might result in NSRS. Chun et al. [60] found that when the true strain is lower than 0.08, tension in the ND and compression in the RD for a strong-textured AZ31 rolled plate can result in NSRS; with increasing strain level, the SRS index *m* increases gradually and turns out to be positive when the true strain exceeds 0.08. Microstructural examination indicates that twinning dominates plastic deformation when the strain is lower than 0.08, while the dislocation slip takes over when the strain is higher than 0.08, where twinning is saturated. Accordingly, it can be concluded that materials exhibit NSRS when plastic strain is dominantly mediated by deformation twinning. Karimpoor et al. [59] also found the twinning-induced NSRS phenomenon in NC Co with a strong basal texture. The NC Mg alloy in the present work has two things in common with the above two materials: strong texture and the same crystal structure (HCP). It is reasonable to infer that enhanced twinning activities at higher strain rates lead to NSRS phenomenon in the NC Mg alloy. This mechanism can well explain those special features aforementioned. First, ageing treatment can result in a grain boundary solute segregation and formation of solute clusters [7], which can suppress twin nucleation [68,69]. Thus, contribution of twinning to plastic strain decreases, while that of the dislocation slip increases, leading to the increase in *m* from −0.030 to −0.016 (see Figure 6). Second, when the loading direction is perpendicular to the longitudinal section, a part of the grains are oriented ins such a way that the basal slip is readily activated and therefore dominates deformation in the low strain rate range, resulting in PSRS (see Figure 7). When LSR exceeds 3 × 10^−2^ s^−1^, twinning is activated and takes over plastic deformation, causing NSRS (see Figure 7). Third, the abrupt decrease in *m* of the cross section at 3 × 10^−1^ s^−1^ might result from the activation of less favored twin variants in addition to the most favored ones, which further enhances twinning activities at higher strain rates (see Figure 7).

### 4.2. Negative Strain Rate Dependence of Creep Displacement

During the plastic deformation of NC metallic materials, dislocations are emitted from GBs, traverse through the grains, and are eventually accumulated at or absorbed by the opposite GBs. The dislocation absorption model established by Carlton et al. [70] suggests that the probability of a dislocation to be absorbed by GB is related to strain rate. The higher the strain rate, the lower the absorption probability. Due to the lower absorption probability, higher LSRs will induce a higher density of dislocations stored in grains. During the holding stage, these stored dislocations can continue to move forward under applied loads, leading to creep. Accordingly, the higher the LSR, the more the stored dislocations, and consequently, the larger the creep displacements during the holding stage. This model well explains the normal strain rate dependence of indentation creep displacements in previous studies on CG and NC materials [17,42,71]. In the present work, with increasing LSR, twinning activities are enhanced, generating more twin boundaries. During the holding stage, these twin boundaries will impede the motion of stored dislocations and shorten their mean glide distances, which therefore reduces creep displacements. The higher the LSR, the more the twin boundaries, and consequently, the smaller the creep displacements. This results in the negative strain rate dependence of indentation creep displacements (see Figure 8). As discussed in Section 4.1, ageing treatment can suppress twinning activities. Consequently, the amount of twin boundaries in the RS+A NC alloy is less than those in RS sample, and the corresponding impeding effect on dislocation motion is weakened, leading to larger creep displacements in the RS+A NC Mg alloy, which is distinctly different from the common sense that ageing can strengthen alloys and therefore improve their creep resistance [72,73].

## 5. Conclusions

The strain rate sensitivity and indentation creep behavior of a bulk nanocrystalline Mg–Gd–Y–Zr alloy were investigated. The main findings were as follows:

1. Nanocrystalline Mg–Gd–Y–Zr alloy exhibits negative strain rate sensitivity. Enhanced twinning activities at higher loading strain rates lead to the decrease in hardness, that is, the negative strain rate sensitivity.
2. Initial unaged nanocrystalline Mg–Gd–Y–Zr alloy creeps via a dislocation viscous glide mechanism due to the solute drag effect, while the creep mechanism of aged alloy turns to a dislocation climb owing to the depletion of solute atoms by segregation and clustering.
3. Indentation creep displacements of nanocrystalline Mg–Gd–Y–Zr alloy exhibit a negative strain rate dependence. Enhanced twinning activities at higher loading strain rates generate more twin boundaries in the alloy, which impede dislocation motion, shorten their mean glide distances, and therefore reduce the creep displacements. Suppressed twinning activities in aged alloy result in larger creep displacements than those in unaged alloy.

## Figures and Tables

**Table 1:** Samples examined in this work.

Samples	Examined Direction	Loading Mode
CG–Ext.	Cross section	CSR-depth
NC–RS	Cross and Longitudinal section	CSR-depth and CSR-load
NC–RS+A	Cross section	CSR-depth

**Table 2:** Comparison between creep stress exponents obtained via conventional and work-of indentation.

Samples	Conventional Approach, *n*	Work-of-Indentation Approach, *n*_cr_
NC–RS	88.9	2.9
NC–RS+A	32.4	7.7

## Data Availability

The data presented in this study are available on request from the corresponding author.
